# Comparative RNA-Seq Analysis of High- and Low-Oil Yellow Horn During Embryonic Development

**DOI:** 10.3390/ijms19103071

**Published:** 2018-10-08

**Authors:** Li Wang, Chengjiang Ruan, Lingyue Liu, Wei Du, Aomin Bao

**Affiliations:** 1 Key Laboratory of Biotechnology and Bioresources Utilization, Ministry of Education, Institute of Plant Resources, Dalian Minzu University, Dalian 116600, China; liwang@dlnu.edu.cn (L.W.); lly19940908@163.com (L.L.); duwei@dlnu.edu.cn (W.D.); 2 Institute of economic forest, Tongliao Academy of Forestry Science and Technology, Tongliao 028000, China; baoaomin@hotmail.com

**Keywords:** yellow horn, gene expression, RNA-seq, oil content, embryonic development

## Abstract

Yellow horn (*Xanthoceras sorbifolium* Bunge) is an endemic oil-rich shrub that has been widely cultivated in northern China for bioactive oil production. However, little is known regarding the molecular mechanisms that contribute to oil content in yellow horn. Herein, we measured the oil contents of high- and low-oil yellow horn embryo tissues at four developmental stages and investigated the global gene expression profiles through RNA-seq. The results found that at 40, 54, 68, and 81 days after anthesis, a total of 762, 664, 599, and 124 genes, respectively, were significantly differentially expressed between the high- and low-oil lines. Gene ontology (GO) enrichment analysis revealed some critical GO terms related to oil accumulation, including acyl-[acyl-carrier-protein] desaturase activity, pyruvate kinase activity, acetyl-CoA carboxylase activity, and seed oil body biogenesis. The identified differentially expressed genes also included several transcription factors, such as, AP2-EREBP family members, B3 domain proteins and C2C2-Dof proteins. Several genes involved in fatty acid (FA) biosynthesis, glycolysis/gluconeogenesis, and pyruvate metabolism were also up-regulated in the high-oil line at different developmental stages. Our findings indicate that the higher oil accumulation in high-oil yellow horn could be mostly driven by increased FA biosynthesis and carbon supply, i.e. a source effect.

## 1. Introduction

Yellow horn (*Xanthoceras sorbifolium* Bunge), belonging to the Sapindaceae family, is an endemic oil-rich shrub widely distributed in northern China and grows well in barren lands with a dry climate. Yellow horn seeds are abundant in oil (55–70%), which is comprised of 85–93% unsaturated fatty acids (FAs), mainly including oleic acid, linoleic acid, gondoic acid and nervonic acid [1,2]. Therefore, this woody oil-bearing tree species has been deemed suitable for cultivation in deserts and arid and semi-arid zones within northern China. With an increasing world population and global demands for healthy edible oils, yellow horn breeding programs aim to select high-oil yielding cultivars in an effort to achieve food security in a sustainable manner.

During the past decade, a number of studies have examined yellow horn seed oil, but have predominantly focused on oil extraction, fatty acid (FA) composition, or use as a biodiesel [1,2,3]. To achieve the maximum amount of oil yield, a better understanding of the mechanisms of oil biosynthesis and the specific molecular pathways involved is necessary. Several studies have attempted to examine yellow horn oil biosynthesis metabolic pathways and have identified some genes found to be associated with oil accumulation [4,5,6]. One study found that the expression of two novel diacylglycerol acyltransferase genes (*XsDGAT1* and *XsDGAT2*) in transgenic *Arabidopsis* led to triacylglycerol (TAG) assembly and increased seed oil production [4]; with the expression patterns of both genes also correlated with oil accumulation in developing yellow horn embryos, presumably via TAG assembly. When studying the molecular mechanisms of oil biosynthesis in yellow horn via functional expressional analysis of *XsSAD* in *Escherichia coli* and *Arabidopsis*, a correlation between *XsSAD* expression and FA accumulation was noted [6]. Furthermore, *XsSAD* was found to determine the synthesis of oleic acid and contributed to unsaturated FA accumulation in yellow horn seeds.

To date, a large number of studies have investigated mechanistic differences in oil biosynthetic pathways between high- and low-oil lines in various oil-producing crops such as oil palm [7], rape seed [8,9,10], soybean [11], sunflower [12], and maize [13,14]. These studies used a variety of approaches, including microarray/gene expression [8,11], proteomics studies [14,15], metabolomic studies [16], enzyme activities [12,17], and microscopic analysis of oil bodies [9] to try to elucidate genes associated with oil content. With the advent of next-generation sequencing (NGS), whole genome-wide expression profiling has become a rapid and efficient approach for identifying key genes and specific metabolic pathways associated with oil yield, especially in plants whose complete genomes have not been fully sequenced. Recently, de novo transcriptome analysis of a pooled yellow horn sample (buds, leaves, flowers and seeds) was performed, which identified pathways and genes related to oil biosynthesis and metabolism [18]. This study serves as a foundation for further studies relating to the metabolic engineering of yellow horn in an effort to increase oil content and modify oil composition. While yellow horn may be a source of genetic determinants for oil accumulation, oil biosynthesis-related gene discovery efforts have been limited, with the molecular mechanisms that modulate oil content still poorly understood.

Herein, RNA-seq was utilized to further characterize the genetic basis of higher oil accumulation in yellow horn. RNA-seq profiles were generated for embryo tissues from high- and low-oil lines at four different developmental stages and compared to identify key genes and pathways contributing to the higher oil content. This approach identified a great number of genes likely to play critical roles in promoting oil accumulation, including several transcription factors, and genes involved in FA biosynthesis, glycolysis/gluconeogenesis, and pyruvate metabolism. The findings presented herein will aid in a further understanding of the processes that contribute to high-oil yields in yellow horn and will provide useful information for future breeding programs.

## 2. Results

### 2.1. Characterizations of Oil Accumulation in the Two Yellow Horn Lines

**Figure 1:**
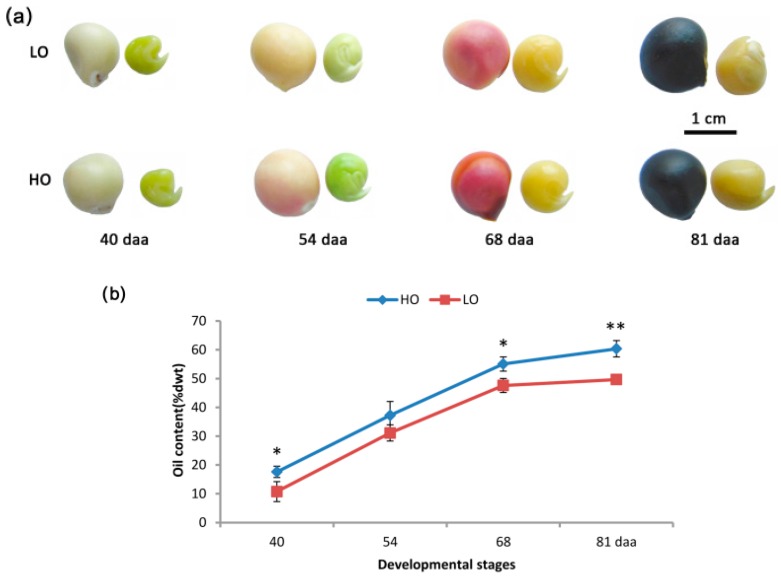
High-oil (HO) and low-oil (LO) yellow horn lines at four developmental stages. (**a**) HO and LO seeds and embryos; (**b**) HO and LO oil content. ** and * indicate significant differences in oil content between the two lines at the same development stage by *t*-test at *p* < 0.01 and *p* < 0.05. Error bars represent the SD for three independent measurements.

The major objective of this study was to identify genes associated with increased oil production in developing embryos. Oil content in the high-oil (HO) line was about 60.36% of the embryo weight, while in the low-oil (LO) line, it was 49.67%. Yellow horn embryos were imaged at four different time points during development (40, 54, 68 and 81 days after anthesis (daa) for comparison (Figure 1a). Additionally, oil content was monitored during the course of development from 40 daa (early embryo stage) to 81 daa (fully matured stage). The HO line oil content exhibited a rapid accumulation of almost 3-fold from 40 daa (17.60 ± 1.99%) to 68 daa (55.07 ± 2.49%; Figure 1b). A similar increase was seen in the LO line, thus indicating that substantial oil accumulation occurs during the early to middle stages of embryo development. However, while similar trends were noted, the HO embryos showed significantly higher oil content than the LO embryos at 40, 68 and 81 daa, with the exception of 54 daa.

### 2.2. Sequencing Statistics and Assembly

A total of 16 cDNA libraries were constructed from HO and LO lines at four development stages, with two biological replicates used for each embryo developmental stage for each line. About 58.77 million pair-end reads of ~150 bp long were obtained for each library. In total, 940,298,242 raw reads were successfully obtained from 16 libraries. After quality trimming, a total of 922,403,070 clean reads remained (98.1% of the raw reads), corresponding to 134.87 gigabyte of sequences. These clean reads had more than 97.78% Q20 bases.

Using the Trinity program [19], all pair-end reads were subsequently assembled de novo into 109,446 transcripts, with an N50 length of 1617 bp and an average length of 914 bp. From these transcripts, a total of 79,915 unigenes (genes) with an N50 length of 1250 bp and an average length of 718 bp were obtained (Supplementary Data 1). Genes that were assembled in silico ranged from 200 to 17,183 bp with a total size of approximately 57.39 Mb (Figure S1). Genes with lengths between 200 and 500 bp were overrepresented and accounted for approximately 61.72% of the total number of genes. A total of 14,310 genes were 500–1000 bp (17.91%) and 16,284 genes were >1000 bp (20.38%).

### 2.3. Function Annotation of Transcriptome Genes

For gene functional annotation, all assembled genes were aligned against multiple databases, including the NCBI non-redundant protein (Nr), the manually annotated and curated protein sequence (Swiss-Prot), the Protein family (Pfam), the euKaryotic Ortholog Groups (KOG), the Gene ontology (GO), and the Kyoto Encyclopedia of Genes and Genomes (KEGG) databases. A total of 38,088 genes (47.66%) were annotated in at least one database (Table 1). Among these, 37,043 (46.35%) had significant matches in the Nr database, while 21,450 (26.84%) showed similarities to proteins in the Swiss-Prot database. Additionally, 18,936 (23.70%) genes were annotated in the GO and 11,984 (15%) in KEGG databases.

### 2.4. Analysis of Differentially Expressed Genes (DEGs) in High- and Low-Oil Yellow Horn During Embryonic Development

**Figure 2:**
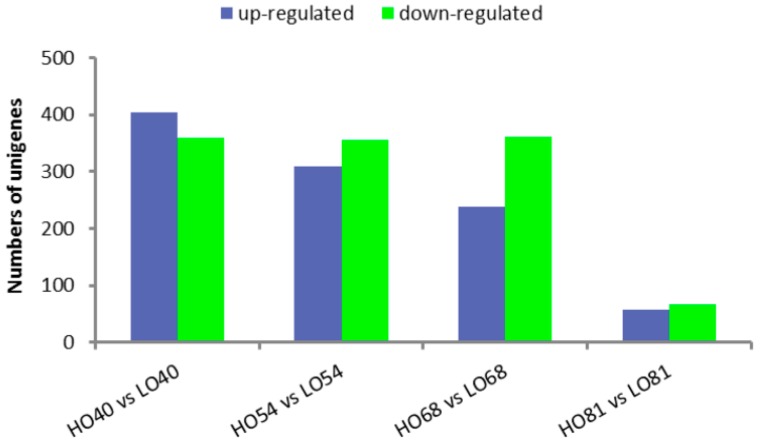
Differentially expressed genes (DEGs) between HO and LO at four development stages. Genes up-regulated (blue) and down-regulated (green) in HO relative to LO at matched developmental stages.

Approximately 81.95–93.72% of the pair-end reads in the 16 libraries were mapped to the yellow horn global transcriptome (Table 2), thus suggesting that the transcriptome is a reliable reference. When comparing the HO40 and LO40 libraries, a total of 762 genes were significantly differentially expressed (Figure 2, Table S1). Subsequent GO enrichment analysis identified 107 GO terms, mainly pertaining to stress response, monolayer-surrounded lipid storage body, translation elongation factor activity, very long-chain FA biosynthetic process and brassinosteroid-mediated signaling pathway (Table S2). 664 DEGs were identified between HO54 and LO54 (Table S3), with 86 GO terms found to be significantly enriched following GO analysis. The enriched GO terms were mainly involved in intermembrane transport, monolayer-surrounded lipid storage body, acyl-[acyl-carrier-protein] desaturase activity, lipid binding and pyruvate kinase activity (Table S4). Furthermore, 599 DEGs were found between HO68 and LO68 (Table S5), with 109 GO terms found to be significantly enriched following GO analysis. The identified GO terms were related to translation, structural constituent of ribosome, acetyl-CoA carboxylase activity, and seed oil body biogenesis (Table S6). Lastly, between HO81 and LO81 (Table S7), the number of DEGs decreased significantly to 124, thus suggesting that little difference of gene expression exists between the two lines during the mature stages. For these DEGs, 52 GO terms were enriched, including brassinosteroid-mediated signaling pathway, cytoplasmic transport, and alcohol dehydrogenase (NADP+) activity (Table S8).

**Figure 3:**
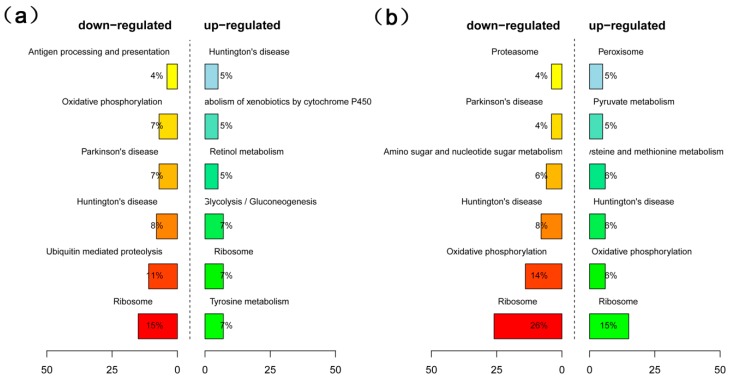

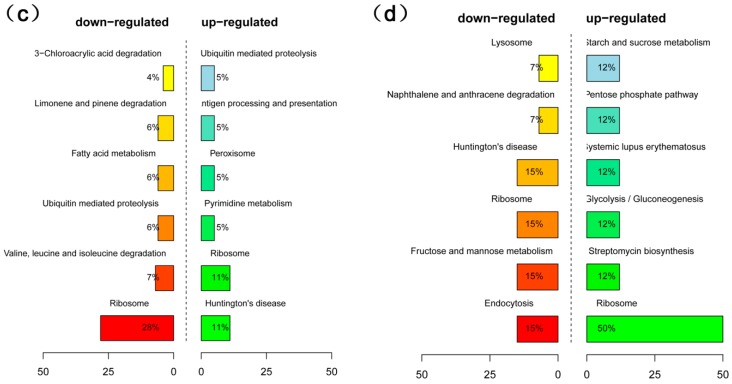
KEGG pathway enrichment analysis based on identified DEGs. KEGG pathway enrichment analysis based on associated DEGs between (**a**) HO40 and LO40, (**b**) HO54 and LO54, (**c**) HO68 and LO68, and (**d**) HO81 and LO81.

For KEGG pathway analysis of the HO40 and LO40 libraries, 123 genes were mapped to 107 pathways (Table S9). The genes that were up-regulated in HO40 relative to LO40 were annotated to pathways associated with glycolysis/gluconeogenesis (7%), ribosome (7%), and tyrosine metabolism (7%), while the HO40 down-regulated genes were associated with ribosome (15%), ubiquitin mediated proteolysis (11%), and oxidative phosphorylation (7%) pathways (Figure 3a, Table S9). At 54 daa, the up-regulated genes in HO54 relative to LO54 were associated with pyruvate metabolism (5%), cysteine and methionine metabolism (6%) and peroxisome (5%) pathways (Figure 3b, Table S10). Furthermore, at 68 daa, the genes that were down-regulated in HO68 relative to LO68 were associated with valine, leucine, and isoleucine degradation (7%) and fatty acid metabolism (5%) pathways (Figure 3c, Table S11). Once the mature stage was reached (81 daa), the up-regulated genes in HO81 were mainly involved in carbohydrate metabolism, such as glycolysis/gluconeogenesis, starch and sucrose metabolism, and the pentose phosphate pathway (Figure 3d, Table S12).

### 2.5. DEGs Related to FA (Fatty Acid) Biosynthesis

**Figure 4:**
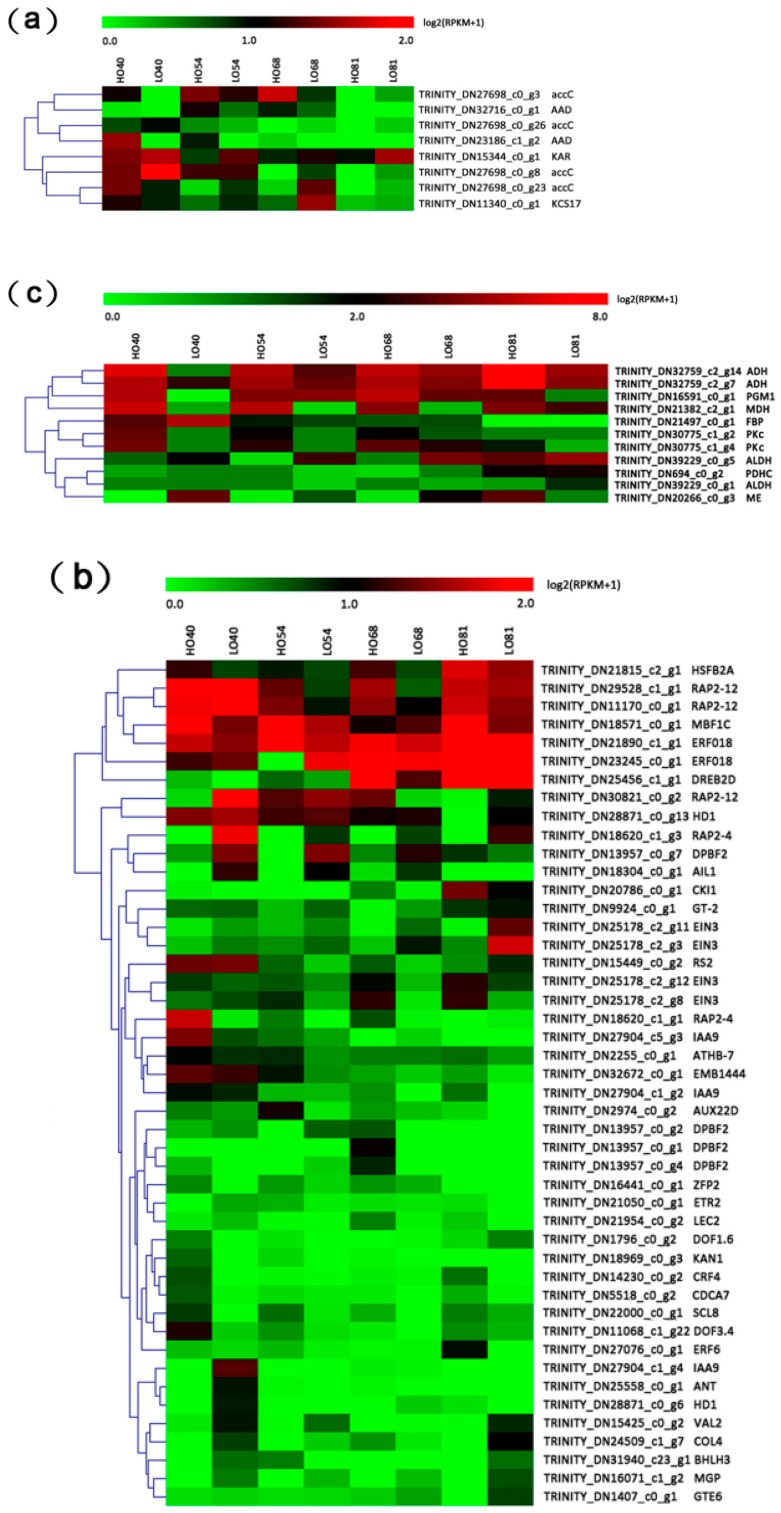
Heatmap diagrams of the expression levels of DEGs between the HO and LO lines at four development stages. (**a**) genes relating to FA biosynthesis; (**b**) transcription factors; (**c**) genes relating to carbon metabolism. Gene expression data were normalized to log2. Red indicates high expression, black indicates intermediate expression, and green indicates low expression.

**Figure 5:**
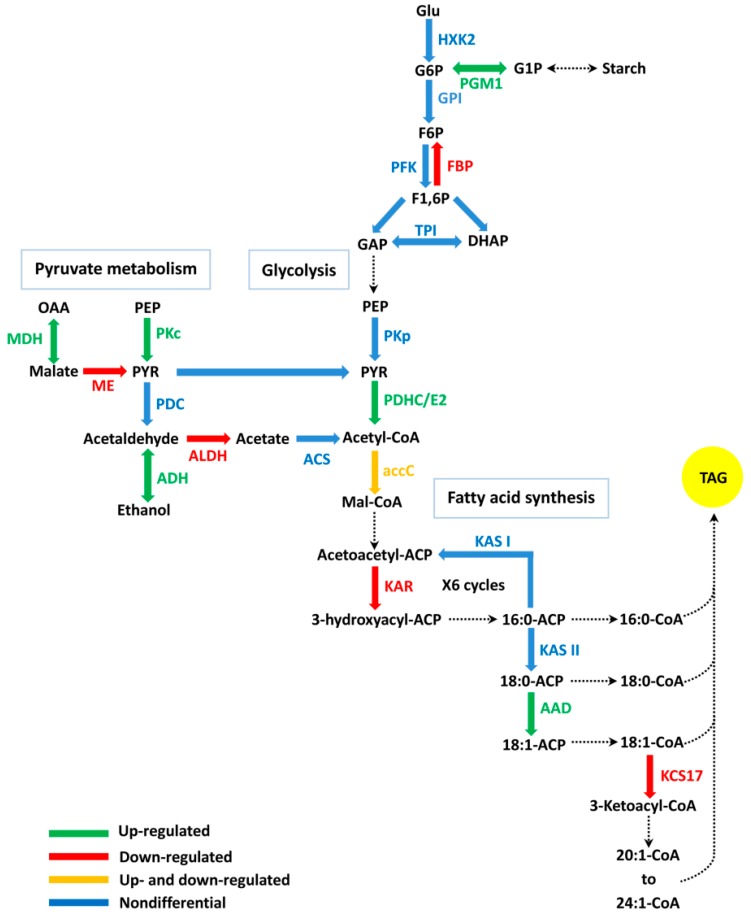
Summary of the DEGs in pathways that are involved directly or indirectly in oil biosynthesis in high-oil yellow horn. Green represents up-regulated genes, red represents down-regulated genes, orange represents both up-regulated and down-regulated genes and blue represents non-differentially expressed genes. Dashed lines indicate multistep pathways. Abbreviations: AAD, acyl-ACP desaturase; accC, acetyl-CoA carboxylase/biotin carboxylase; ACS, acetyl-CoA synthetase; ADH, alcohol dehydrogenase; ALDH, aldehyde dehydrogenase; DHAP, dihydroxyacetone-3-biphosphate; FBP, fructose-1,6-bisphosphatase; F1,6P, fructose 1,6-biphosphate; F6P, fructose-6-phosphate; HXK2, hexokinase2; GAP, glyceraldehyde 3-phosphate; Glu, glucose; G6P, glucose-6-phosphate; G1P, glucose-1-phosphate; GPI, glucose phosphate isomerase; KAR, 3-Ketoacyl ACP reductase; KAS I, 3-ketoacyl-ACP synthase I; KAS II, 3-ketoacyl-ACP synthase II; KCS17, 3-ketoacyl-CoA synthase 17; Mal-CoA, malonyl-CoA; MDH, malate dehydrogenase; ME, NADP-dependent malic enzyme; PDC, pyruvate decarboxylase; PDHC/E2, pyruvate dehydrogenase complex/E2 component; PFK, phosphofructose kinase; PGM1, phosphoglucomutase; PKc, cytosolic pyruvate kinase; PKp, plastidial pyruvate kinase; PEP, phosphoenolpyruvate; PYR, pyruvate; OAA, oxaloacetate; TAG, triacylglycerol; TPI, triosephosphate isomerase.

In this study, several DEGs relating to FA biosynthetic processes (GO:0006633) were identified at both the early and middle stages of embryonic development (40, 54, and 68 daa) (Table S13). However, the GO terms were not significantly enriched in this study. The profiles of eight DGEs involved in FA biosynthesis during embryonic development were examined (Figure 4a) and included acetyl-CoA carboxylase/biotin carboxylase (*accC*), 3-Ketoacyl ACP reductase (*KAR*), acyl-ACP desaturase (*AAD*), and 3-ketoacyl-CoA synthase 17 (*KCS17*) (Figure 5). During the early stage of embryonic development (40 daa), with the exception of one down-regulated *accC* gene, two *accC* genes (TRINITY_DN27698_c0_g3 and TRINITY_DN27698_c0_g23) were significantly up-regulated in HO40 as compared to LO40; while at 68 daa, most of the *accC* genes were significantly down-regulated in HO68. Additionally, the expression of two *AAD* genes (TRINITY_DN32716_c0_g1 and TRINITY_DN23186_c1_g2) was up-regulated in HO relative to LO during embryonic development, with significant up-regulation noted at 54 daa (Figure 5). Surprisingly, the gene (TRINITY_DN15344_c0_g1) encoding KAR was found to be down-regulated in HO at 54 daa and *KCS17* (TRINITY_DN11340_c0_g1) was also down-regulated in HO at 68 daa (Figure 5).

### 2.6. Differentially Expressed Transcription Factors (TFs)

Considering the functional importance of transcription factors, 46 differentially expressed TFs were identified between HO and LO during embryonic development (Figure 4b, Table S14). GO classifications assigned them to the regulation of transcription (GO:0006355), sequence-specific DNA binding transcription factor activity (GO:0003700), positive regulation of transcription (GO:0045893), and seed oil body biogenesis (GO:0010344). A total of six genes encoding RAP2-12, RAP2-4, ERF018, DREB2D, and ERF6 (AP2-EREBP family members) were found to be up-regulated in HO relative to LO at various time points. Of these AP2-EREBP TFs, only *RAP2-4* (TRINITY_DN18620_c1_g1) was found to be up-regulated in HO at 40 daa, which is when FAs begin to accumulate relatively rapidly; while *RAP2-12* (TRINITY_DN29528_c1_g1 and TRINITY_DN11170_c0_g1), *ERF018* (TRINITY_DN21890_c1_g1), and *DREB2D* (TRINITY_DN25456_c1_g1) were mainly up-regulated during the middle stage of embryonic development. Furthermore, two C2C2-Dof TFs, *DOF3.4* (TRINITY_DN11068_c1_g22) and *DOF1.6* (TRINITY_DN1796_c0_g2), were also significantly up-regulated in HO at 40 daa. Interestingly, two B3 TFs, *LEC2* (TRINITY_DN21954_c0_g2) and *VAL2* (TRINITY_DN15425_c0_g2), displayed a different expression pattern, with *LEC2* significantly up-regulated in HO relative to LO at 68 daa; while *VAL2* was significantly down-regulated in HO at 40 and 54 daa, thus suggesting different regulatory roles on oil accumulation during embryonic development.

### 2.7. DEGs Related to Glycolysis/Gluconeogenesis and Pyruvate Metabolic Pathways

During embryonic development, a total of 11 DGEs were found to be involved in glycolysis/gluconeogenesis and pyruvate metabolic pathways (PATHWAY: ko00010 and ko00620), with most being up-regulated in HO relative to LO (Figure 4c, Table S15). Among these DGEs, two genes (TRINITY_DN30775_c1_g2 and TRINITY_DN30775_c1_g4) encoding cytosolic pyruvate kinase (PKc) were up-regulated in HO at 40 and 54 daa when compared to time matched LO samples (Figure 5). Moreover, pyruvate dehydrogenase complex/E2 component (*PDHC/E2*; TRINITY_DN694_c0_g2) and phosphoglucomutase (*PGM1*; TRINITY_DN16591_c0_g1) were also significantly up-regulated in HO at 40 and 81 daa; while plastid fructose-1,6-bisphosphatase (*FBP*; TRINITY_DN21497_c0_g1) was down-regulated in HO at 40 daa (Figure 5). Additionally, within the pyruvate metabolic pathway, one gene (TRINITY_DN21382_c2_g1) putatively encoding malate dehydrogenase (MDH) was significantly up-regulated in HO at 54 and 68 daa, while the gene encoding NADP-dependent malic enzyme (ME; TRINITY_DN20266_c0_g3) was down-regulated at 40 daa (Figure 5).

### 2.8. qPCR Validation of DEGs

**Figure 6:**
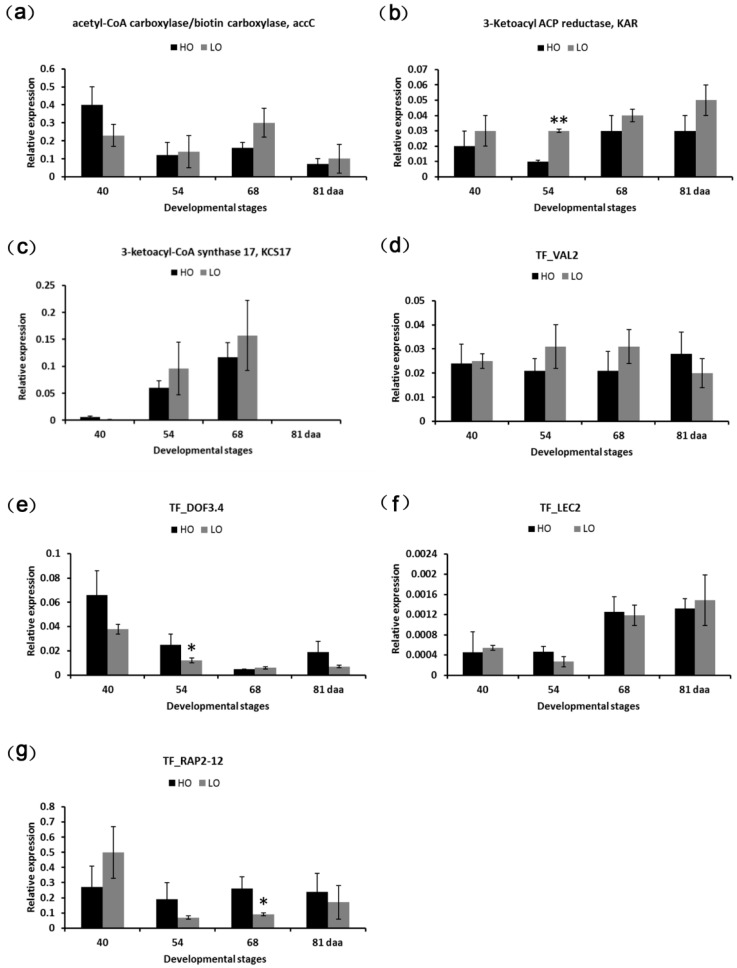
qPCR expressional profiles of 7 DEGs identified via RNA-seq. The *X*-axis represents sampling time points corresponding to the four embryonic development stages (40, 54, 68, and 81 daa). The *Y*-axis represents the relative level of expression. Genes are (**a**) *accC* (Acetyl-CoA carboxylase/biotin carboxylase); (**b**) *KAR* (3-Ketoacyl ACP reductase); (**c**) *KCS17* (3-ketoacyl-CoA synthase 17); (**d**) TF_*VAL2*; (**e**) TF_*DOF3.4*; (**f**) TF_*LEC2*; and (**g**) TF_*RAP2-12*. ** and * indicate significant differences in HO relative to LO at the same development stage by *t*-test at *p* < 0.01 and *p* < 0.05. Error bars represent the SD for three biological replicates.

To validate our RNA-seq sequencing data, seven DEGs of interest were selected across different developmental stages based on the RNA-seq results. The genes included *accC* (TRINITY_DN27698_c0_g3), *KAR* (TRINITY_DN15344_c0_g1), *KCS17* (TRINITY_DN11340_c0_g1), *DOF3.4* (TRINITY_DN11068_c1_g22), *VAL2* (TRINITY_DN15425_c0_g2), *RAP2-12* (TRINITY_DN29528_c1_g1), and *LEC2* (TRINITY_DN21954_c0_g2). The validation experiments were carried out using the embryo samples collected at the same developmental stages as those that were used in the RNA-seq experiment. The qPCR results showed that *accC*, *DOF3.4*, *LEC2*, and *RAP2-12* had higher expression levels in HO relative to LO at different developmental stages; while *KAR*, *KCS17*, and *VAL2* were down-regulated in HO at most of the developmental stages (Figure 6). These findings were nearly consistent with the respective RNA-seq data. However, qPCR analysis of *accC* showed no differential expression between HO68 and LO68, while the RNA-seq data showed a significant up-regulation in HO68 relative to LO68.

## 3. Discussion

### 3.1. The Function of FA Biosynthesis on Oil Accumulation

Extensive studies have suggested that FA synthesis may be an important regulatory step in oil production within vascular plants [20,21,22,23]. Acetyl-CoA carboxylase (ACCase) is considered the rate-limiting enzyme controlling the flux of carbon into FAs [24,25]. ACCase consists of four subunits, with three being nuclear-encoded subunits (biotin carboxylase, biotin carboxyl carrier protein and carboxyl transferase α-subunit) and one being a plastid-encoded subunit (carboxyl transferase β-subunit). The higher levels of *accC* and carboxyl transferase β-subunit (*accD*) expression were found to be correlated with higher productivity and oil content in oil palm [26]. Furthermore, the plastid-encoded subunit seems to be crucial to plastid ACCase accumulation and promotes increased oil content [27]. However, the expression profiles generated herein identified two genes encoding accC (a nuclear-encoded subunit), with significantly higher expression levels noted in HO compared to LO at 40 daa, implying that the increased expression levels of *accC* genes are correlated with increased oil content in the yellow horn embryos examined. It is proposed that two up-regulated *accC* genes may promote FA biosynthesis at the early stage of embryonic development, which further promote oil accumulation in developing yellow horn embryos, resulting in higher oil content in the high-oil line (Figure 5). In contrast to two above *accC* genes, one *KAR* gene was shown to be down-regulated in HO compared to LO at 54 daa, which was different from what was observed in high oil-yielding palm previously [7]. In the plastid, the extension of FA from malonyl-ACP is iteratively catalyzed by a type II FA synthase (FAS) system consisting of 3-ketoacyl-ACP synthase (KAS), KAR, hydroxyacyl-ACP dehydrogenase (HAD), and enoyl-ACP reductase (EAR) [28]. Given that the *KAR* gene was considerably down-regulated, while the total oil content was increased in HO relative to LO, we speculate that *KAR* transcripts or enzyme quantities must have already been elevated to promote FA biosynthesis in HO yellow horn.

Acyl-ACP desaturase is a plastid-localized soluble desaturase that catalyzes the desaturation of stearic acid (18:0) to oleic acid, which plays crucial role in determining the ratio of saturated to unsaturated FAs. Recently, *AAD* expression was found to strongly correlate with the oleic acid, unsaturated FA, and total FA levels in developing yellow horn embryos [6]. Furthermore, our study identified two *AAD* genes that were significantly up-regulated in HO relative to LO at 54 daa, suggesting that *AAD* expression is also correlated with total oil content in the yellow horn embryos examined. Therefore, the enhanced expression of *AAD*, together with *accC*, at the early and early-middle stages of embryonic development could contribute to elevated oil accumulation by promoting increased FA supply in HO yellow horn (Figure 5). On the basis of these results, we hypothesize that higher oil accumulation in yellow horn embryo is driven by the flux towards FA generated by *accC* and *AAD*, i.e. a source effect, without concerted transcriptional regulation of TAG assembly genes.

### 3.2. Role of Transcriptional Regulation in Oil Accumulation

Transcription factors are important regulators which can regulate the development, maturation, oil biosynthesis and deposition of plant seeds [29,30], but little is known about the transcriptional regulation of oil biosynthesis in yellow horn seeds. Recently, by homologous annotation of the transcripts, several TFs genes related to oil biosynthesis were detected in yellow horn [18]. However, the key TFs that regulate oil accumulation remain unknown. In the present study, 46 differentially expressed TFs were identified between HO and LO during embryonic development, mainly including AP2-EREBP family members, C2C2-Dof proteins and B3 domain proteins. It is worth noting that, of AP2-EREBP family members, the expression level of *DREB2D* was higher in HO compared to that in LO during embryonic development. A DREB-like transcription factor gene *GmDREBL* from soybean was found to enhance the seed lipid content in the transgenic *Arabidopsis* through up-regulation of the master regulatory gene *WRI1* and other genes associated with FA biosynthesis [31]. Considering the increased expression levels of several *ACCase* and *AAD* genes in the HO line, we speculate that the DREB2D transcription factor may participate in the regulation of oil accumulation by promoting the expression of FA biosynthesis-related genes in yellow horn embryos. Moreover, GmDREBL can directly bind to the promoter region of *WRI1* to activate its expression in plants [31]. WRI1, a member of the AP2-EREBP family, is now considered to be a ubiquitous regulator of oil biosynthesis in higher plants [32]. However, *WRI1* genes presented herein were not significantly differentially expressed between the HO and LO lines during embryonic development. Whether *DREB2D* regulates oil accumulation by activating the expression of *WRI1* in yellow horn embryos remains to be investigated in the future.

Plant embryo development is regulated by a network of various TFs that mainly include AFL (for ABI3/FUS3/LEC2) B3 and VAL B3 proteins [33,34]. LEC2 activates the embryo maturation program, while the closely related VAL2 suppresses embryo maturation and growth by shutting down the AFL network before germination. Double mutants of *VAL1* and *VAL2* produced seedling expressing embryo specific genes, AFL clade genes, and accumulated high levels of seed storage compounds [35]. Furthermore, the *LEC* genes were found to be involved in regulation of FA biosynthesis and storage lipid deposition during embryonic development [36]. In *Arabidopsis*, senescence-induced *LEC2* expression led to a three-fold increase in TAG levels in leaves [37]. Interestingly, the present study identified the *LEC2* gene that was significantly up-regulated in HO yellow horn at 68 daa, whereas the gene encoding VAL2 was found to be down-regulated in HO relative to LO at 40 and 54 daa. Collectively, these results indicated that *LEC2* played a positive role in seed oil biosynthesis and deposition during embryonic development, and the decreased expression of *VAL2* observed herein could contribute to the derepression of the AFL B3 network, resulting in the induction of TFs associated with the maturation phase, and ultimately leading to higher oil accumulation.

### 3.3. The Relation Between Carbon Metabolism and Oil Accumulation

Glycolysis, which is central to carbon metabolism, converts sugars into precursors for protein and FA biosynthesis, while also producing ATP by substrate level phosphorylation. The glycolytic pathway serves as a principal source for carbon skeletons and provides the reductions needed for lipid biosynthesis. Pyruvate, a direct carbon precursor for FA biosynthesis, can be produced from phosphoenolpyruvate (PEP) via glycolysis, which is catalyzed by PK, or a small quantity of pyruvate can also be synthesized from malate by ME [38,39]. When performing de novo FA biosynthesis in plastids, pyruvate is further converted to acetyl-CoA by the PDHC [40]. PDHC consists of four subunits: E1-α, E1-β, E2, and E3 [40]. Previous studies in several oil-producing crops have confirmed a link between increased *PK* or *PDHC* expression and higher oil accumulation [41]. It has also been shown that in high-oil palms, *PDHC* was up-regulated compared to the low-oil group [7]. Similarly, in this study, *PKc* and *PDHC/E2* (downstream genes involved in glycolysis) were found to be significantly up-regulated in HO compared to LO yellow horn at 40 and 54 daa. Interestingly, upstream genes such as *PGM1* and *FBP* that are involved in carbon metabolism also showed significantly altered expression between the two lines, suggesting carbon source partitioning between starch and oil accumulation in developing yellow horn embryos (Figure 5). Hence, considering the functions of these genes and their expression profiles, it seems possible that the up-regulation of *PK* and *PDHC*, which are key enzymes in the production of precursors for FA synthesis, as well as the altered expression of *PGM1* and *FBP*, could increase the supply of pyruvate, which leads to greater oil production through the FA biosynthesis machinery.

Malate has also been shown to contribute to oil biosynthesis by acting as a carbon unit and a reductant source [42]. Within pyruvate metabolism, MDH catalyzes the bi-directional interconversion of malate and oxaloacetate (OAA). In this study, the most notable expressional difference associated with pyruvate metabolism was seen in *MDH*, with the HO gene being 154 and 37 times higher than in LO at 54 and 68 daa. These findings indicate that the production of malate or oxaloacetate, which can be subsequently converted into pyruvate in the cytosol or plastid, is accelerated in the HO line (Figure 5).

Taken together, these results suggest that glycolysis is up-regulated in the HO line compared to the LO line during embryonic development, resulting in the increase of carbon supply in HO yellow horn. Pyruvate metabolism also provides carbon units (malate and oxaloacetate) for FA biosynthesis. These findings imply a stronger funneling of carbon towards pyruvate/acetyl-CoA, a precursor for FA biosynthesis leading to increased oil accumulation, in the HO line compared to the LO line. Thus, increased FA biosynthesis, together with carbon supply, is likely crucial for the elevated oil accumulation in the HO line, i.e., a source effect.

In summary, we report the first comparative analysis of global gene expression between high- and low-oil yellow horn. Our analysis identified a great number of genes likely to play critical roles in promoting oil accumulation, including several transcription factors, and genes involved in FA biosynthesis, glycolysis/gluconeogenesis and pyruvate metabolism. However, further studies are clearly required to validate the function of these genes related to oil accumulation in yellow horn. Overall, these resources provide a strong genetic basis for metabolic engineering research to enhance oil content of yellow horn, and may also provide some reference for researching the closely oil producing species. Our study also allows breeders to identify candidate genes involved in high oil synthesis, and develop new molecular markers based on polymorphism of the regulatory genes related to oil accumulation. This will pave the way for marker-assisted selection of yellow horn and expedite the breeding of yellow horn with a higher oil content.

## 4. Materials and Methods

### 4.1. Plant Material

Yellow horn blossoms in early May and its fruits ripen in late July. All embryo tissues were obtained from yellow horn in field trials located in Naiman Banner, Inner Mongolia, China (121.04° E, 43.21° N). Two yellow horn lines (NM1203 and NM1003) with similar genetic backgrounds were selected as the HO and LO classification groups, respectively. This determination was based on the embryo average yearly oil content as assessed by bunch analysis over a 3-year period. The two lines were established using systematic breeding of natural populations and selected by massal selection. The Dice coefficient between the HO and LO lines was calculated at 0.8899 based on 23 simple sequence repeat (SSR) markers. Four inflorescences were open-pollinated and fruit bunches were harvested at 40, 54, 68, and 81 daa in 2016. The yellow horn fruits collected at each sampling were randomized, with the fresh embryo tissues collected, snap frozen, and stored at −80 °C.

### 4.2. Oil Content Analysis

The seeds from the HO and LO lines were dried to a consistent weight at 80 °C and then pulverized in a ball mill. The seed oil was extracted from the embryos at 40, 54, 68, and 81 daa as previously described [43]. Each sample was analyzed three times, with the data reported as a mean ± SD.

### 4.3. RNA-Seq Library Preparation and Sequencing

Total RNA was extracted from yellow horn embryos using a Spin Column Plant Total RNA Extraction kit (Sangon Biotech, Shanghai, China) and contaminating DNA was digested with RNase-Free DNase I (Tiangen, Beijing, China) according to the manufacturer’s instructions. RNA degradation and contamination levels were assessed on 1% agarose gels. RNA quantification and purity was determined using a Nanodrop ND-2000 spectrophotometer (Thermo Scientific, Waltham, MA, USA) and RNA integrity was assessed using an Agilent Bioanalyzer 2100 system (Agilent Technologies, Santa Clara, CA, USA). RNA-Seq libraries were constructed according to the Illumina manufacturer’s instructions (Illumina, San Diego, CA, USA). Briefly, poly (A)-mRNA was isolated from 6 μg of total RNA per sample using oligo-dT molecules attached to magnetic beads. The mRNA was then fragmented into smaller pieces using divalent cations under an elevated temperature. The cleaved RNA fragments were used for first-strand cDNA synthesis using random hexamers and reverse transcriptase. Second-strand cDNA synthesis was subsequently performed using buffer, dNTPs, RNase H and DNA polymerase I. The double stranded cDNA were purified using AMPure XP beads (Beckman Coulter Genomics, Danvers, MA, USA) and subjected to end repair process, adenylation and then ligated to Illumina multiplex barcode adapters. The adaptor ligated cDNAs were size selected with AMPure XP beads and subjected to PCR amplification to enrich the adapter-ligated fragments, which were further purified using AMPure XP beads. At last, library quality was assessed on the Agilent Bioanalyzer 2100 system (Agilent Technologies, Santa Clara, CA, USA). The clustering of the index-coded samples was performed on a cBot Cluster Generation System using the HiSeq PE Cluster Kit v4 (Illumina, San Diego, CA, USA) following the manufacturer’s instructions. After cluster generation, RNA-Seq libraries were sequenced using the Illumina HiSeq 4000 instrument at LC Sciences (Hangzhou, China) and generated 150 bp paired-end reads. Two independent replicates were used for each embryo developmental stage for each line. All raw data have been submitted to the NCBI BioProject, and the accession number is PRJNA493982.

### 4.4. Quality Control

FastQC (version 0.10.1) [44] was used to check the quality of raw reads generated by Illumina. To obtain high-quality clean reads, the raw reads were first trimmed by removing adaptor sequences using the Cutadapt software (version 1.11) [45]. Each sequence was then scanned for low quality regions, and if a 6 bp sliding window showed an average quality score less than 20, the read would be truncated at that position using the fqtrim software (version 0.9.7) [46]. All reads in which unknown bases were more than 5% and reads with lengths less than 100 bp were also removed using the fqtrim software. All downstream analyses were therefore based on high-quality clean data.

### 4.5. De novo Assembly and Annotation

After quality filtering, the 16 obtained libraries were pooled for the de novo assembly of the global transcriptome using the short reads assembly program Trinity (version 2.2.0) (Kmer = 25; min_kmer_cov = 10; min_contig_length = 200) [19]. Pair-end reads were first assembled into contigs according to their overlap regions. Then, the contigs were clustered, and all clean reads were mapped back to the contigs. With paired-end reads, it is possible to detect the contigs from the same transcript as well as the sequences between these contigs. Finally, the contigs were further assembled, and the assembled sequences that could not be extended on either end were defined as transcripts. The longest transcripts in the cluster units derived from a given gene were defined as genes. All assembled genes were matched and annotated against the Nr, Swiss-Prot, Pfam, KOG, and KEGG databases using BLASTX with an *E*-value ≤ 1 × 10^−10^. GO functional classifications were obtained using the hypergeometric distribution algorithm according to molecular function, biological process and cellular component ontologies (Available online: http://www.geneontology.org/). KEGG pathway annotation was also performed using the online KEGG Automatic Annotation Server (KAAS) [47,48].

### 4.6. Reads Mapping to the Reference Genome

The de novo transcriptome data was selected as the reference. The obtained pair-end reads from the 16 libraries were aligned to the reference genome, and obtained from the annotation information of each sample using the bowtie algorithm with default parameters in the RSEM software (version 1.2.31) [49].

### 4.7. Quantification of Gene Expression Level and Differential Expression Analysis

RSEM software was used to estimate raw read counts from the alignments, which can be unambiguously assigned to genomic features (transcripts) for each sample. Following the alignment, raw counts of individual genes were normalized to reads per kilobase of exon model per million mapped reads (RPKM) [50] to obtain the relative levels of expression. Genes were defined as the unique or shared expressed transcripts among the HO and LO lines based on the RPKM value (RPKM > 0). Differential expression analysis was performed using the DESeq R package [51] for comparisons among samples based on the read count for each gene at different development stages. The resulting P values were adjusted using the Benjamini and Hochberg’s approach to control the false discovery rate (FDR) [52]. FDR ≤ 0.05 and an absolute value of log2 Ratio ≥ 1 were used as the threshold for significant differential expression.

To correct potential scale effect of gene expression and avoid working with negative expression values, the RPKM values of the expressed genes were normalized by the log2 method. Clustering analysis of DEGs was conducted with the normalized RPKM values using the hclust command in *R* language [53], and the heatmap was generated in R using the heatmap.2 function in the gplots CRAN library.

### 4.8. GO and KEGG Enrichment Analysis of DEGs

To study the biological significance of DEGs, GO and KEGG enrichment analyses were conducted using a Fisher’s exact test, with the significance threshold corrected to a *p*-value < 0.05.

### 4.9. Quantitative Real-Time PCR (qPCR) Validation

Total RNA was extracted as described above. For first-strand cDNA synthesis, PrimeScript™ RT Master Mix (TaKaRa Biotech Co., Dalian, China) was used according to the manufacturer’s instructions. The expression levels of the selected genes were analyzed by qPCR using an Applied Biosystems 7500 Real-Time PCR System. Specific primers were designed using Primer 3 software (Table S16). The qPCR reactions were performed in 20 μL volumes containing 10 μL of 2× SYBR Premix Ex Taq II (Tli RNaseH Plus; TaKaRa Biotech Co., Dalian, China), 1.6 μL of primer mix (0.4 μM final concentration per primer), 1 μL of cDNA templates, 0.4 μL of 50× ROX Reference Dye II, and 7 μL of sterile water. The thermal program for the qPCR reaction was 95 °C for 30 s, followed by 40 cycles of 95 °C for 5 s and 60 °C for 34 s. The specificity of each amplification reaction was verified by melt curve analysis. No template controls were included for each primer pair and three biological and experimental replicates were performed for each sample. Relative expression levels of target genes were analyzed using the 2^−Δ*C*t^ method [54], with samples normalized against the reference gene actin (ACT) as previously reported [6].

## Figures and Tables

**Table 1:** Annotations of the yellow horn transcriptome.

Annotated Databases	Number of Annotated Genes	Percentage of Annotated Genes
Nr	37,043	46.35%
SwissProt	21,450	26.84%
Pfam	26,318	32.93%
KOG	28,946	36.22%
GO	18,936	23.70%
KEGG	11,984	15.00%
Annotated in at least one database	38,088	47.66%
Total number of genes	79,915	100%

**Table 2:** Summary of clean reads aligned to the reference transcriptome for each library.

Sample Name	Total No. of Clean Reads	Reads Mapped	Percentage of Mapped Reads (%)
LO40_1	54,222,626	47,575,480	87.74%
LO40_2	63,290,894	53,054,604	83.83%
LO54_1	51,724,078	48,476,883	93.72%
LO54_2	59,978,660	56,140,038	93.60%
LO68_1	58,729,700	53,863,346	91.71%
LO68_2	62,4351,74	54,673,650	87.57%
LO81_1	62,510,098	57,732,856	92.36%
LO81_2	47,836,828	44,564,451	93.16%
HO40_1	53,006,640	43,436,912	81.95%
HO40_2	64,749,242	54,235,860	83.76%
HO54_1	59,699,584	53,801,599	90.12%
HO54_2	61,692,916	57,458,341	93.14%
HO68_1	51,553,508	47,005,432	91.18%
HO68_2	62,726,966	58,733,804	93.63%
HO81_1	56,526,196	52,826,069	93.45%
HO81_2	51,719,960	47,510,223	91.86%

## Supplementary Materials

Supplementary materials can be found at http://www.mdpi.com/1422-0067/19/10/3071/s1.

## Abbreviations

GO	Gene Ontology
KOG	euKaryotic Ortholog Groups
KEGG	Kyoto Encyclopedia of Genes and Genomes
HO	High-oil
LO	Low-oil
daa	Days after anthesis
DEGs	Differentially expressed genes
accC	Acetyl-CoA carboxylase/biotin carboxylase
FA	Fatty acid
KAR	3-Ketoacyl ACP reductase
KAS	3-Ketoacyl-ACP synthase
HAD	Hydroxyacyl-ACP dehydrogenase
EAR	Enoyl-ACP reductase
AAD	Acyl-ACP desaturase
KCS17	3-Ketoacyl-CoA synthase 17
PKc	Cytosolic pyruvate kinase
PDHC/E2	Pyruvate dehydrogenase complex/E2 component
PGM1	Phosphoglucomutase
FBP	Fructose-1,6-bisphosphatase
MDH	Malate dehydrogenase
ME	NADP-dependent malic enzyme
TFs	Transcription factors
ALDH	Aldehyde dehydrogenase
ADH	Alcohol dehydrogenase
PEP	Phosphoenolpyruvate
OAA	Oxaloacetate
PYR	Pyruvate
TAG	Triacylglycerol

## References

[1] Zhang S., Zu Y.G., Fu Y.J., Luo M., Liu W., Li J., Efferth T. (2010). Supercritical carbon dioxide extraction of seed oil from yellow horn (*Xanthoceras sorbifolia* Bunge.) and its anti-oxidant activity. Bioresour. Technol..

[2] Ruan C.J., Yan R., Wang B.X., Mopper S., Guan W.K., Zhang J. (2017). The importance of yellow horn (*Xanthoceras sorbifolia*) for restoration of arid habitats and production of bioactive seed oils. Ecol. Eng..

[3] Li J., Fu Y.J., Qu X.J., Wang W., Luo M., Zhao C.J., Zu Y.G. (2012). Biodiesel production from yellow horn (*Xanthoceras sorbifolia* Bunge.) seed oil using ion exchange resin as heterogeneous catalyst. Bioresour. Technol..

[4] Guo H.H., Wang T.T., Li Q.Q., Zhao N., Zhang Y., Liu D., Hu Q., Li F.L. (2013). Two novel diacylglycerol acyltransferase genes from *Xanthoceras sorbifolia* are responsible for its seed oil content. Gene.

[5] Guo H.H., Li Q.Q., Wang T.T., Hu Q., Deng W.H., Xia X.L., Gao H.B. (2014). *XsFAD2* gene encodes the enzyme responsible for the high linoleic acid content in oil accumulated in *Xanthoceras sorbifolia* seeds. J. Sci. Food Agric..

[6] Zhao N., Zhang Y., Li Q.Q., Li R.F., Xia X.L., Qin X.W., Guo H.H. (2015). Identification and expression of a stearoyl-ACP desaturase gene responsible for oleic acid accumulation in *Xanthoceras sorbifolia* seeds. Plant Physiol. Biochem..

[7] Wong Y.C., The H.F., Mebus K., Ooi T.E.K., Kwong Q.B., Koo K.L., Ong C.K., Mayes S., Chew F.T., Appleton D.R. (2017). Differential gene expression at different stages of mesocarp development in high- and low-yielding oil palm. BMC Genom..

[8] Fu S.X., Cheng H., Qi C. (2009). Microarray analysis of gene expression in seeds of *Brassica napus* planted in Nanjing (altitude: 8.9 m), Xining (altitude: 2261.2 m) and Lhasa (altitude: 3658 m) with different oil content. Mol. Biol. Rep..

[9] Hu Z., Wang X., Zhan G., Liu G., Hua W., Wang H. (2009). Unusually large oilbodies are highly correlated with lower oil content in *Brassica napus*. Plant Cell Rep..

[10] Guan M., Li X., Guan C. (2012). Microarray analysis of differentially expressed genes between *Brassica napus* strains with high- and low-oleic acid contents. Plant Cell Rep..

[11] Wei W.H., Chen B., Yan X.H., Wang L.J., Zhang H.F., Cheng J.P., Zhou X.A., Sha A.H., Shen H. (2008). Identification of differentially expressed genes in soybean seeds differing in oil content. Plant Sci..

[12] Troncoso-Ponce M.A., Garcés R., Martínez-Force E. (2010). Glycolytic enzymatic activities in developing seeds involved in the differences between standard and low oil content sunflowers (*Helianthus annuus* L.). Plant Physiol. Biochem..

[13] Tsaftaris A.S., Scandalios J.G. (1983). Genetic analysis of isocitrate lyase enzyme activity levels in maize lines selected for high or low oil content. J. Hered..

[14] Liu Z., Yang X., Fu Y., Zhang Y., Yan J., Song T., Rocheford T., Li J. (2009). Proteomic analysis of early germs with high-oil and normal inbred lines in maize. Mol. Biol. Rep..

[15] Loei H., Lim J., Tan M., Lim T.K., Lin Q.S., Chew F.T., Kulaveerasingam H., Chung M.C. (2013). Proteomic analysis of the oil palm fruit mesocarp reveals elevated oxidative phosphorylation activity is critical for increased storage oil production. J. Proteome Res..

[16] Teh H.F., Neoh B.K., Hong M.P., Low J.Y., Ng T.L., Ithnin N., Thang Y.M., Mohamed M., Chew F.T., Yusof H.M. (2013). Differential metabolite profiles during fruit development in high-yielding oil palm mesocarp. PLoS ONE.

[17] Settlage S., Kwanyuen P., Wilson R. (1998). Relation between diacylglycerol acyltransferase activity and oil concentration in soybean. J. Am. Oil Chem. Soc..

[18] Liu Y.L., Huang Z.D., Ao Y., Li W., Zhang Z. (2013). Transcriptome analysis of yellow horn (*Xanthoceras sorbifolia* Bunge): A potential oil-rich seed tree for biodiesel in China. PLoS ONE.

[19] Grabherr M.G., Haas B.J., Yassour M., Levin J.Z., Thompson D.A., Amit I., Adiconis X., Fan L., Raychowdhury R., Zeng Q. (2011). Full-length transcriptome assembly from RNA-Seq data without a reference genome. Nat. Biotech..

[20] Bourgis F., Kilaru A., Cao X., Ngando-Ebongue G.F., Drira N., Ohlrogge J.B., Arondel V. (2011). Comparative transcriptome and metabolite analysis of oil palm and date palm mesocarp that differ dramatically in carbon partitioning. Proc. Natl. Acad. Sci. USA.

[21] Troncoso-Ponce M.A., Kilaru A., Cao X., Durrett T.P., Fan J., Jensen J.K., Thrower N.A., Pauly M., Wilkerson C., Ohlrogge J.B. (2011). Comparative deep transcriptional profiling of four developing oilseeds. Plant J..

[22] Venglat P., Xiang D., Qiu S., Stone S.L., Tibiche C., Cram D., Alting-Mees M., Nowak J., Cloutier S., Deyholos M. (2011). Gene expression analysis of flax seed development. BMC Plant Biol..

[23] Dussert S., Guerin C., Andersson M., Joet T., Tranbarger T.J., Pizot M., Sarah G., Omore A., Durand-Gasselin T., Morcillo F. (2013). Comparative transcriptome analysis of three oil palm fruit and seed tissues that differ in oil content and fatty acid composition. Plant Physiol..

[24] Nikolau B.J., Ohlrogge J.B., Wurtele E.S. (2003). Plant biotin-containing carboxylases. Arch. Biochem. Biophys..

[25] Andre C., Haslam R.P., Shanklin J. (2012). Feedback regulation of plastidic acetyl-CoA carboxylase by 18:1-acyl carrier protein in *Brassica napus*. Proc. Natl. Acad. Sci. USA.

[26] Nakkaew A., Chotigeat W., Eksomtramage T., Phongdara A. (2008). Cloning and expression of a plastid-encoded subunit, beta-carboxyltransferase gene (*accD*) and a nuclear-encoded subunit, biotin carboxylase of acetyl-CoA carboxylase from oil palm (*Elaeis guineensis* Jacq.). Plant Sci..

[27] Cui Y., Liu Z., Zhao Y., Wang Y., Huang Y., Li L., Wu H., Xu S., Hua J. (2017). Overexpression of heteromeric *GhACCase* subunits enhanced oil accumulation in upland cotton. Plant Mol. Biol. Rep..

[28] Ohlrogge J., Browse J. (1995). Lipid biosynthesis. Plant Cell.

[29] Palaniswamy S.K., James S., Sun H., Lamb R.S., Davuluri R.V., Grotewold E. (2006). AGRIS and AtRegNet. a platform to link cis-regulatory elements and transcription factors into regulatory networks. Plant Physiol..

[30] Niu J., Chen Y., An J., Hou X., Cai J., Wang J., Zhang Z., Lin S. (2015). Integrated transcriptome sequencing and dynamic analysis reveal carbon source partitioning between terpenoid and oil accumulation in developing *Lindera glauca* fruits. Sci. Rep..

[31] Zhang Y.Q., Lu X., Zhao F.Y., Li Q.T., Niu S.L., Wei W., Zhang W.K., Ma B., Chen S.Y., Zhang J.S. (2016). Soybean GmDREBL increases lipid content in seeds of transgenic *Arabidopsis*. Sci. Rep..

[32] Ma W., Kong Q., Arondel V., Kilaru A., Bates P.D., Thrower N.A., Benning C., Ohlrogge J.B. (2013). *WRINKLED1*, a ubiquitous regulator in oil accumulating tissues from *Arabidopsis* embryos to oil palm mesocarp. PLoS ONE.

[33] Suzuki M., Wang H.H., McCarty D.R. (2007). Repression of the *LEAFY COTYLEDON 1/B3* Regulatory Network in Plant Embryo Development by *VP1/ABSCISIC ACID INSENSITIVE 3-LIKE* B3 Genes. Plant Physiol..

[34] Suzuki M., McCarty D.R. (2008). Functional symmetry of the B3 network controlling seed development. Curr. Opin. Plant Biol..

[35] Veerappan V., Chen N., Reichert A.I., Allen R.D. (2014). HSI2/VAL1 PHD-like domain promotes H3K27 trimethylation to repress the expression of seed maturation genes and complex transgenes in *Arabidopsis* seedlings. BMC Plant Biol..

[36] Vanhercke T., El Tahchy A., Liu Q., Zhou X.R., Shrestha P., Divi U.K., Ral J.P., Mansour M.P., Nichols P.D., James C.N. (2014). Metabolic engineering of biomass for high energy density: Oilseed-like triacylglycerol yields from plant leaves. Plant Biotechnol. J..

[37] Kim H.U., Lee K.R., Jung S.J., Shin H.A., Go Y.S., Suh M.C., Kim J.B. (2015). Senescence-inducible LEC2 enhances triacylglycerol accumulation in leaves without negatively affecting plant growth. Plant Biotechnol. J..

[38] Kang F., Rawsthorne S. (1996). Metabolism of glucose-6-phosphate and utilization of multiple metabolites for fatty acid synthesis by plastids from developing oilseed rape embryos. Planta.

[39] Alonso A.P., Goffman F.D., Ohlrogge J.B., Shachar-Hill Y. (2007). Carbon conversion efficiency and central metabolic fluxes in developing sunflower (*Helianthus annuus* L.) embryos. Plant J..

[40] Lin M., Behal R., Oliver D.J. (2003). Disruption of *plE2*, the gene for the E2 subunit of the plastid pyruvate dehydrogenase complex, in *Arabidopsis* causes an early embryo lethal phenotype. Plant Mol. Biol..

[41] Li R.J., Wang H.Z., Mao H., Lu Y.T., Hua W. (2006). Identification of differentially expressed genes in seeds of two near-isogenic *Brassica napus* lines with different oil content. Planta.

[42] Baud S., Lepiniec L. (2010). Physiological and developmental regulation of seed oil production. Prog. Lipid Res..

[43] Shockey J.M., Gidda S.K., Chapital D.C., Kuan J.C., Dhanoa P.K., Bland J.M., Rothstein S.J., Mullen R.T., Dyer J.M. (2006). Tung tree DGAT1 and DGAT2 have nonredundant functions in triacylglycerol biosynthesis and are localized to different subdomains of the endoplasmic reticulum. Plant Cell.

[44] FastQC. http://www.bioinformatics.babraham.ac.uk/projects/fastqc/.

[45] Martin M. (2011). Cutadapt removes adapter sequences from high-throughput sequencing reads. Embnet J..

[46] fqtrim. http://ccb.jhu.edu/software/fqtrim/index.shtml.

[47] Aoki-Kinoshita K.F., Kanehisa M. (2007). Gene annotation and pathway mapping in KEGG. Methods Mol. Biol..

[48] Moriya Y., Itoh M., Okuda S., Yoshizawa A.C., Kanehisa M. (2007). KAAS: An automatic genome annotation and pathway reconstruction server. Nucleic Acids Res..

[49] Li B., Dewey C.N. (2011). RSEM: Accurate transcript quantification from RNA-Seq data with or without a reference genome. BMC Bioinform..

[50] Mortazavi A., Williams B.A., McCue K., Schaeffer L., Wold B. (2008). Mapping and quantifying mammalian transcriptomes by RNA-Seq. Nat. Meth..

[51] Anders S., Huber W. (2010). Differential expression analysis for sequence count data. Genome Biol..

[52] Benjamini Y., Hochberg Y. (1995). Controlling the false discovery rate: A practical and powerful approach to multiple testing. J. R. Stat. Soc. B.

[53] R: A Language and Environment for Statistical Computing. https://www.R-project.org.

[54] Schmittgen T.D., Livak K.J. (2008). Analyzing real-time PCR data by the comparative C_T_ method. Nat. Protoc..

