# Supplementary material for: Comparative RNA-Seq Analysis of High- and Low-Oil Yellow Horn During Embryonic Development
Source: Int J Mol Sci. 2018 Oct 8;19(10):3071. doi: 10.3390/ijms19103071 (PMC6212864; doi:10.3390/ijms19103071)
Supplement: Supplementary file 1 [file ijms-19-03071-s001.zip › ijms-355635 Supplementary for final check/Supplementary File18_Table S16 Primers used in qPCR for validating DEGs.docx]

**Table S5.** Primers used in qPCR for validating DEGs.

| Gene | Gene ID | Primer sequence 5’→3’ forward/reverse | Amplicon size (bp) |
| --- | --- | --- | --- |
| Actin |  | TACCGAGGCACCATTAAATCCC/ AAGGTCCAAACGAAGAATAGCA | 226 |
| accC | TRINITY_DN27698_c0_g3 | CACGACTGATAGCAGCAGATAG/ CATAGACAAGGACGCACTTCA | 119 |
| KAR | TRINITY_DN15344_c0_g1 | TCAGTTGCCAAGAAACCTACA/CTTGGATGGGAAGACAGAAGAG | 111 |
| KCS17 | TRINITY_DN11340_c0_g1 | GTTAGACGAGCTGGAGAAGAAC/ CTGCTTGAAGTGTTACCAAACC | 93 |
| DOF3.4 | TRINITY_DN11068_c1_g22 | GGAGGAGCAAATGAGAAGGAA/ CCAACCATCAACCACTCTCTAC | 107 |
| VAL2 | TRINITY_DN15425_c0_g2 | GCGCGATGTCTCTTCTTTCT/ GCCAATGTAATGTGTGCCTAAC | 95 |
| LEC2 | TRINITY_DN21954_c0_g2 | GGTTTGGTGTCAGGAGTAGAAG/ CTCTGACCAACTTTGGACTCTC | 109 |
| RAP2-12 | TRINITY_DN29528_c1_g1 | CGGTGATTGCCCATCAAGATA/ GGAGGAAGACAACGAAGTACAG | 100 |
