# Supplementary figures and images for: Comparative RNA-Seq Analysis of High- and Low-Oil Yellow Horn During Embryonic Development
Source: Int J Mol Sci. 2018 Oct 8;19(10):3071. doi: 10.3390/ijms19103071 (PMC6212864; doi:10.3390/ijms19103071)

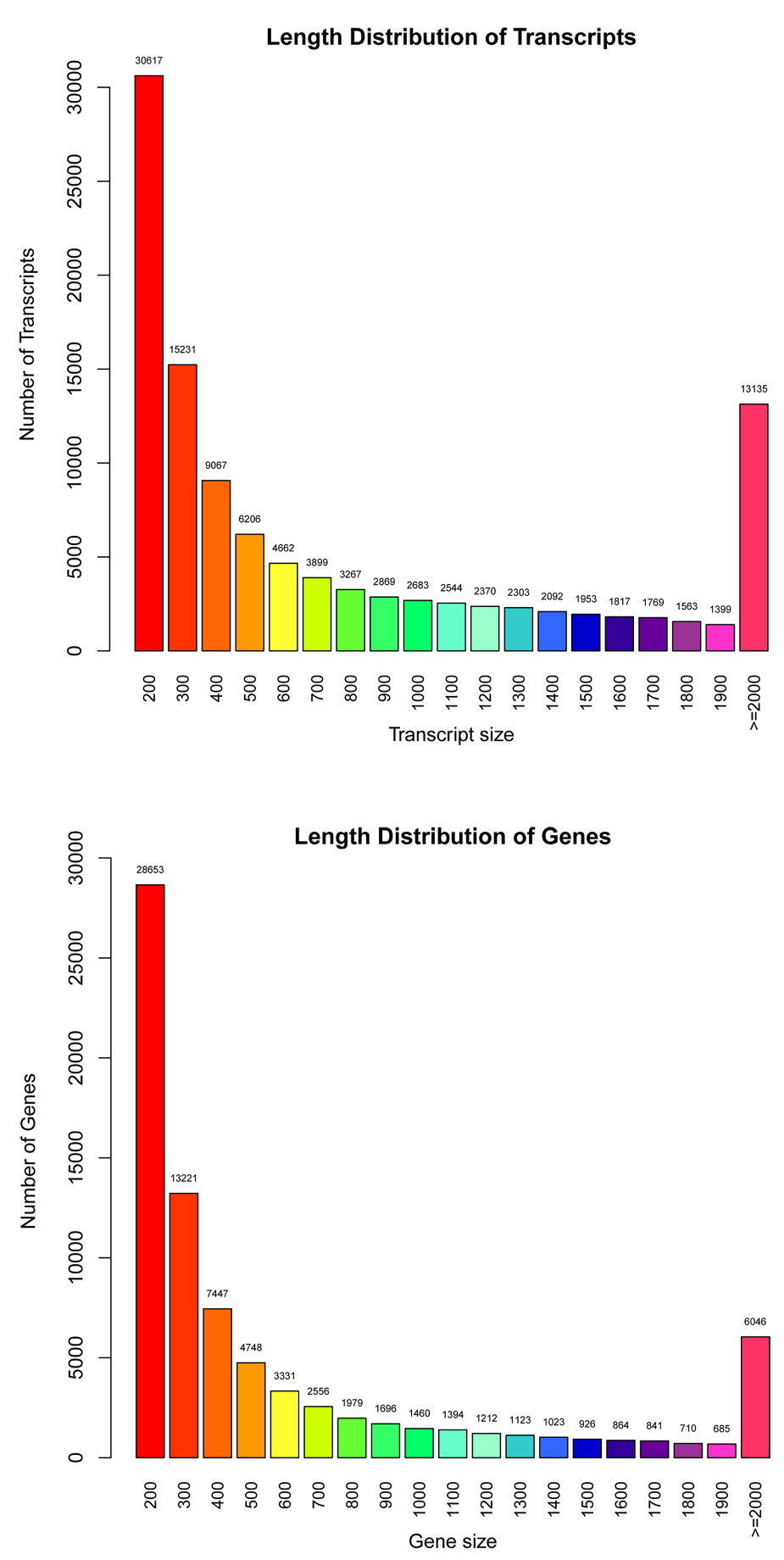


**Figure S1.** Length distribution of all transcripts and genes.

Supplement: Supplementary file 1 [file ijms-19-03071-s001.zip › ijms-355635 Supplementary for final check/Supplementary File2-Figure S1.docx]
